# A 0.5-Mbp deletion on bovine chromosome 23 is a strong candidate for stillbirth in Nordic Red cattle

**DOI:** 10.1186/s12711-016-0215-z

**Published:** 2016-04-18

**Authors:** Goutam Sahana, Terhi Iso-Touru, Xiaoping Wu, Ulrik Sander Nielsen, Dirk-Jan de Koning, Mogens Sandø Lund, Johanna Vilkki, Bernt Guldbrandtsen

**Affiliations:** Department of Molecular Biology and Genetics, Center for Quantitative Genetics and Genomics, Aarhus University, 8830 Tjele, Denmark; Natural Resources Institute Finland, 31600 Jokioinen, Finland; Danish Agricultural Advisory Service, 8200 Århus N, Denmark; Department of Animal Breeding and Genetics, Swedish University of Agricultural Sciences, 75007 Uppsala, Sweden

## Abstract

**Background:**

A whole-genome association study of 4631 progeny-tested Nordic Red dairy cattle bulls using imputed next-generation sequencing data revealed a major quantitative trait locus (QTL) that affects birth index (BI) on *Bos taurus* autosome (BTA) 23. We analyzed this QTL to identify which of the component traits of BI are affected and understand its molecular basis.

**Results:**

A genome-wide scan of BI in Nordic Red dairy cattle detected major QTL on BTA6, 14 and 23. The strongest associated single nucleotide polymorphism (SNP) on BTA23 was located at 13,313,896 bp with $- \log_{10} ({\text{p}}) = 50.63$. Analyses of component traits showed that the QTL had a large effect on stillbirth. Based on the 10 most strongly associated SNPs with stillbirth, we constructed a haplotype. Among this haplotype’s alleles, HAP_QTL_ had a large negative effect on stillbirth. No animals were found to be homozygous for HAP_QTL_. Analysis of stillbirth records that were categorized by carrier status for HAP_QTL_ of the sire and maternal grandsire suggested that this haplotype had a recessive mode of inheritance. Illumina BovineHD BeadChip genotypes and genotype intensity data indicated a chromosomal deletion between 12.28 and 12.81 Mbp on BTA23. An independent set of Illumina Bovine50k BeadChip genotypes identified a recessive lethal haplotype that spanned the deleted region.

**Conclusions:**

A deleted region of approximately 500 kb that spans three genes on BTA23 was identified and is a strong candidate QTL with a large effect on BI by increasing stillbirth.

**Electronic supplementary material:**

The online version of this article (doi:10.1186/s12711-016-0215-z) contains supplementary material, which is available to authorized users.

## Background

Previously, the molecular causes of genetic defects in cattle were mainly detected by examining dead or malformed calves [1–9]. The recent availability of high-throughput genotyping platforms and next-generation sequencing technologies has substantially accelerated the discovery of lethal genetic factors [10–14]. However, polymorphisms that are not associated with characteristic phenotypic manifestations, such as physically malformed calves, remain difficult to identify. A complementary approach is quantitative trait locus (QTL) mapping. For example, a QTL with a large effect on fertility in Nordic Red dairy cattle was first reported on *Bos taurus* autosome (BTA) 12 [15]. The molecular basis for this QTL was subsequently identified as a 660-kb deletion that causes embryonic death [16]. A whole-genome association scan in Nordic Red dairy cattle detected a major QTL for birth index (BI) on BTA23. BI is a composite index that describes the contribution of additive genetic effects to a calf’s genetic potential to be born.

In this study, we analyzed two component traits that contribute to BI (i.e. stillbirth and calving ease) for the QTL on BTA23. Our objectives were: (1) to identify which component traits are affected by the BI QTL on BTA23, and (2) to understand its molecular basis.

## Methods

Animal Care and Use Committee approval was not required for this study because the phenotype and genotype data used were routinely collected as part of a breeding program, and no live animal experiments were performed.

### Animals and traits

The study was carried out on three populations of Nordic Red dairy cattle (RDC) from Denmark (RDCDNK), Finland (RDCFIN) and Sweden (RDCSWE). A total of 4631 progeny-tested bulls with breeding values for BI and its component traits and genotype information were used for QTL mapping.

As dependent traits, we analyzed the de-regressed breeding values (DRP) for calving-related traits. We scanned the breeding values for BI to identify QTL. BI is a compound index that predicts a sire’s total direct additive genetic effect on calving by combining direct (calf) effects of stillbirth (SB) in the first (SBF) and later (SBL) calvings and calving ease (CE) in the first (CEF) and later (CEL) calvings. Calf size (CS) is not included in the prediction of BI by the Nordic Cattle Genetic Evaluation (NAV; http://www.nordicebv.info). However, birth weight is positively correlated with calving assistance/difficulty [17]. Therefore, we analyzed CS in the first (CSF) and later (CSL) calvings. Calves that were alive or dead within 24 h after birth were recorded as 1 or 0 (for SB), respectively. Farmers subjectively assessed and classified CE into two categories in Sweden and four categories in Denmark and Finland) and CS into four categories but this was recorded only in Denmark. We analyzed de-regressed breeding values [18] that were produced as part of the routine breeding value evaluation by the NAV. For further information on the recording and genetic evaluation for direct calving traits, see [19] and http://www.nordicebv.info.

### Genotyping of bulls

All bulls were genotyped by using the BovineSNP50 BeadChip (Illumina, San Diego, CA) version 1 or 2 and genomic DNA extracted from whole blood or semen. Quality control (QC) to select single nucleotide polymorphisms (SNPs) were as follows: a minimum call rate of 85 % for samples, and exclusion of SNPs with a call rate less than 95 %, a minor allele frequency (MAF) less than 0.5 % and a significant deviation from Hardy–Weinberg proportions (HWP; *p* < 10^−5^). After QC, 43,415 SNPs were retained for analyses. Genomic positions of the SNPs were based on the UMD3.1 Bovine Genome Assembly [20].

### Imputation to high-density (HD) and full-genome sequence

The 50 k SNPs were imputed to full sequence in two steps. First, 50 k genotypes for bulls were imputed to HD genotypes by using the IMPUTE2 software with default parameters, except for the effective population size (N_e_) = 100 [21]. The reference population with HD genotypes consisted of 2036 bulls (902 Holstein, 735 Nordic Red and 399 Danish Jersey). The same QC parameters that were used for the 50 k chip were used for HD data. After QC, 648,219 SNPs remained for the HD chip.

In the second imputation step, using whole-genome sequencing (WGS) data from 242 dairy cattle as a reference, 12,322 bulls from three breeds (6032 Holstein, 1645 Jersey and 4645 Nordic Red) imputed to HD genotypes were further imputed to the full-sequence level by using Beagle software [22]. Sequences for the reference population used for imputed animals consisted of WGS data that were obtained by Aarhus University [23, 24] and by the 1000 Bull Genome Project *run2* [14]. Chromosomes were divided into windows of about 20,000 consecutive SNPs, with an overlap of 250 SNPs at each end to minimize imputation errors at each end of the windows. For details on next-generation sequencing data and imputation steps, see [23].

Beagle v3.3.2 [22] was used to pre-phase reference data and to impute from imputed HD genotypes to the full-sequence variants. All SNPs with an imputation certainty (R^2^) value less than 0.9 were removed. A total of 8,938,927 SNPs remained that were distributed across the 29 bovine autosomes [25].

### Statistical models for association analysis

First, a WGS scan was performed using a sire model without considering relationships between individuals, except for the sires and their sons. Based on the results from this scan, we selected a region on BTA23 for further analysis with an animal model that considered relationships between all individuals, using both single-marker and haplotype-based analyses.

#### Sire model for the whole-genome scan

A SNP-by-SNP analysis was carried out, in which each SNP was tested for association with the phenotype (de-regressed breeding value). The following linear mixed model (LMM) was used to estimate SNP effects:

$$y_{ij} = \mu + bx_{ij} + s_{i} + e_{ij} ,$$

where *y*_*ij*_ is the de-regressed breeding value of the *j*th son from the half-sib (sire) family *i*; *μ* is the general mean; *b* is the allelic substitution effect; and *x*_*ij*_ (ranging from 0 to 2) is the allelic dose of the *j*th individual for the SNP obtained by imputation. *s*_*i*_ is the random effect of the *i*th half-sib family, assumed to be normally distributed as $s_{i} \sim N\left( {0,\sigma_{s}^{2} } \right)$, where *σ*_*s*_^2^ is the sire variance. *e*_*ij*_ is the random residual of son *j* from the half-sib family *i* and is assumed to be normally distributed as ${\mathbf{e}} \sim N({\mathbf{0}}, {\mathbf{W}}^{ - 1} \sigma_{e }^{2}$), where ${\mathbf{e}}$ is the vector of random residuals (*e*_*i*_); $\sigma_{e }^{2}$ is the error variance; and ${\mathbf{W}}$ is a diagonal matrix where the diagonal elements are weights of the DRP. The weight of the *i*th animal was estimated by *w*_*i*_ = *r*_*i*_^2^/(1 − *r*_*i*_^2^), where *r*_*i*_^2^ is the reliability of the *ij*th animal’s DRP. Values of *r*_*i*_^2^ that were higher than 0.98 were set to 0.98 to avoid having excessively large weights for sires with large numbers of progeny records.

All statistical analyses were conducted by using the software DMU [26]. The null hypothesis *H*_0_: $b = 0$ was tested with a two-sided *t* test. A SNP was considered to have a significant association with a trait if the $- \log_{10} ({\text{p}})$ was higher than 8.25 (after Bonferroni multiple-testing correction for 8,938,927 simultaneous tests), corresponding to a nominal genome-wide significance level of $p = 0.05$.

#### LMM analysis of the target region on BTA23

LMM analysis was used for association analyses of the target region on BTA23 for BI and its component traits (Table 1). Association between the SNP and phenotype was assessed by a single-locus regression analysis of the phenotype onto the allelic dosage for each SNP separately, using an LMM [27] as follows:

$$y_{j} = \mu + bx_{j} + u_{j} + e_{j} ,$$

where *y*_*j*_ is the phenotype (DRP) for the *j*th bull; *μ* is the overall mean; *b* is the allelic substitution effect; and *x*_*j*_ (ranging from 0 to 2) is the allelic dose of the *j*th individual for the SNP. *u*_*j*_ is the random polygenic effect with a joint multivariate normal distribution, ${\mathbf{u}} \sim N\left({{\mathbf{0}}, {\mathbf{A}} \sigma_{u }^{2} } \right)$, where **u** is the vector of polygenic effects (*u*_*i*_); ${\mathbf{A}}$ is the additive genetic relationship matrix; and $\sigma_{u }^{2}$ is the polygenic variance. *e*_*j*_ is the random residual for the *j*th animal distributed as described in the sire model.

**Table 1:** Most significantly associated SNPs with direct calving traits obtained from linear mixed model analyses for the target region on chromosome 23

Trait	Top associated SNP	MAF	Allele substitution effect	SE	*p* value
BI	Chr23:13,313,896	0.059	−8.76	0.45	4.79e−80
SBF	Chr23:13,313,896	0.059	−7.35	0.45	8.09e−58
SBL	Chr23:13,313,896	0.059	−10.53	0.44	1.00e−120
CSF	Chr23:17,631,540	0.160	1.55	0.19	1.17e−16
CSL	Chr23:17,631,540	0.160	1.60	0.18	6.27e−19
CEF	Chr23:17,631,540	0.160	−1.86	0.21	3.34e−19
CEL	Chr23:17,581,154	0.145	−2.52	0.24	1.26e−25

Suffix F and L are for first and later calvings

Effects and SE are given in breeding index units

*BI* birth index, *SB* stillbirth, *CE* calving ease, *CS* calf size, *MAF* minor allele frequency, *SE* standard error

The model was fitted by restricted maximum likelihood (REML) using the software DMU [26], which provided estimates of the fixed effects and their standard errors. Testing for the effect of a marker was done by using a two-sided t-test against a null hypothesis of *H*_0_: $b = 0$, with a Bonferroni correction for 8,938,927 multiple testing ($- \log_{10} \left( {\text{p}} \right) = 8.25$) similarly to the sire model.

#### Random haplotype model

Haplotypes for the 10 SNPs that were most significantly associated in the LMM analyses and located between 13,259,463 and 13,354,932 bp on BTA23 were extracted from Beagle’s WGS imputation output (Table 2). We used a LMM that included random polygenic effects and random effects of the haplotype, i.e. random haplotype model (RHM) following [28]. The RHM model was:

$$y_{j} = \mu + q_{h1j} + q_{h2j} + u_{j} + e_{j} ,$$

where *q*_*h*1*j*_ and *q*_*h*2*j*_ are random effects of the two haplotypes (one each from the sire and dam) carried by the *j*th individual, assumed to be normally distributed as $q_{hij} \sim N\left( {0,\sigma_{h}^{2} } \right)$, where *σ*_*h*_^2^ is the variance of haplotype effects. Other terms in the RHM were the same as described for the LMM.

**Table 2:** Top 10 most significant SNPs selected to construct haplotypes for haplotype-based association mapping

SNP	MAF	Allele substitution effect	SE	*p* value
Chr23:13,259,463	0.062	−10.15	0.43	4.11e−115
Chr23:13,260,528	0.062	−10.15	0.43	4.05e−115
Chr23:13,260,771	0.062	−10.15	0.43	4.02e−115
Chr23:13,261,303	0.062	−10.15	0.43	4.03e−115
Chr23:13,264,527	0.062	−10.15	0.43	4.14e−115
Chr23:13,269,718	0.062	−10.15	0.43	3.48e−115
Chr23:13,270,817	0.068	−10.22	0.44	2.39e−115
Chr23:13,271,582	0.062	−10.15	0.43	3.48e−115
Chr23:13,313,896	0.059	−10.56	0.44	1.00e−120
Chr23:13,354,932	0.066	−9.84	0.42	3.30e−113

Results are for calf survival at later lactations

Effects and SE are given in breeding value index units

*MAF* minor allele frequency, *SE* standard error

The significance of the haplotype substitution effect was assessed by a likelihood ratio test that compared the RHM model to a null model that included mean, polygenic effect and random error terms, but no haplotype effects. Under *H*_0_, the test statistic has a *χ*^2^ distribution with one degree of freedom. The analysis was performed in the DMU software package [26]. A haplotype with a large negative predicted effect on BI, i.e. HAP_QTL_ was identified as the haplotype that carried the putative causal polymorphism for the QTL. The above LMM analysis was repeated with individual counts of HAP_QTL_ that were added as a fixed regression effect. Carrier status of each bull with respect to HAP_QTL_ was predicted for further analysis, as described below.

### Analysis of calf survival as a function of haplotype carrier status of the sire and maternal grandsire

Recorded pregnancies were classified into types according to the carrier status of the sire and the maternal grandsire for HAP_QTL_. For each pregnancy, we recorded whether the calf was alive or dead 24 h after birth. A recessive lethal mutation can be tested by comparing outcomes from mating types, because there is a 25 % probability that a pregnancy for which both the sire and dam are carriers will result in affected calves. A LMM was applied to test the effect of mating type on calf survival at birth.

Four classes of matings were defined according to the HAP_QTL_ carrier status, i.e. (I) noncarrier sire mated to the daughter of a noncarrier maternal grandsire; (II) noncarrier sire mated to the daughter of a carrier maternal grandsire; (III) carrier sire mated to the daughter of a noncarrier maternal grandsire; and (IV) carrier sire mated to the daughter of a carrier maternal grandsire. Stillbirths for heifers (first calving) and cows (later calvings) were analyzed separately. A total of 3,932,927 calving records from Denmark, Finland and Sweden were analyzed, with 2,785,085 records for mating type I, 536,614 for mating type II, 512,714 for mating type III and 98,514 for mating type IV. Numbers of records in each category are in Table 3.

**Table 3:** Number of observations (N) and calf survival rates (Survival) (%) for mating types according to genotypes of the sire (S) and maternal grandsire (MGS) for the 500-kb deletion

Mother	Mating type								Total
	S-NC × MGS-NC (I)		S-NC × MGS-C (II)		S-C × MGS-NC (III)		S-C × MGS-C (IV)		
	N	Survival	N	Survival	N	Survival	N	Survival	
DNK-heifer	57,165	94.3	1971	94.9	2014	94.6	73	93.2	61,223
DNK-cow	84,679	96.9	2551	96.7	3904	96.9	176	94.3	91,310
FIN-heifer	535,406	94.1	120,843	93.6	105,221	93.0	21,459	88.3	782,929
FIN-cow	805,528	96.3	178,770	96.0	178,395	95.3	36,778	90.4	1,199,471
SWE-heifer	496,779	95.8	53,709	95.3	44,785	95.7	3250	90.7	598,523
SWE-cow	828,052	96.7	97,536	96.5	81,253	96.4	8373	91.9	1,199,471
Total	2,785,085	95.9	536,614	95.6	512,714	95.3	98,514	90.5	3,932,927

*C* carrier, *NC* non carrier, *S* sire, *MGS* maternal grand sire, *FIN* Finland, *SWE* Sweden, *DNK* Denmark

The fitted mixed model included parity and month of insemination (by year) as fixed effects and the maternal grandsire as a random effect:

$$y_{ijkl} = \mu + p_{j} + t_{k} + m_{l} + u_{i} + e_{ijkl} ,$$

where *y*_*ijkl*_ is an indicator of calf survival (0 for survival and 1 for stillbirth); *p*_*j*_ is the effect of parity; *t*_*k*_ is the effect of month and year of insemination; and *m*_*l*_ is the effect of mating type. Vector ${\mathbf{u}}$ of random grandsire effects *u*_*i*_ is assumed to be normally distributed ${\mathbf{u}} \sim N\left( {{\mathbf{0}},\sigma_{g}^{2} {\mathbf{A}}_{s} } \right)$, where ${\mathbf{A}}_{s}$ is the additive genetic relationship among sires of dams derived from the pedigree. ${\mathbf{e}}$ is a vector of random individual error terms $e_{ijkl}$ and is assumed to be normally distributed ${\mathbf{e}} \sim N\left( {{\mathbf{0}}, {\mathbf{I}}\sigma_{e}^{2} } \right)$. Assuming HWP, the expected proportion of conceptuses that are homozygous for HAP_QTL_ is equal to 1/4 of the probability that both parents are carriers, corresponding to 0, 0, $\frac{p}{{4\left( {1 + p} \right)}}$, and $\frac{{\left( {1 + p} \right)}}{{4\left( {2 + p} \right)}}$ for mating types I, II, III, and IV, respectively, where *p* is the frequency of the causative allele [16].

### Search for chromosomal deletions within the QTL for stillbirth

A recessive lethal mutation can be caused by a chromosomal deletion [29, 30]. We searched for chromosomal deletions at the QTL using two approaches: (1) deviation from HWP, and (2) loss of genotype intensity based on SNP array data.

#### Deviation from HWP

If the recessive lethal effect is due to a chromosomal deletion, then an excess of homozygotes will be called for the SNPs that are located within the deleted region [16]. Imputed HD SNP genotypes in the QTL region were investigated for deviations from HWP. Among the imputed SNP genotypes, we tested for deviations of their frequencies from HWP by using a *χ*^2^ distribution with one degree of freedom. For SNPs that deviated from HWP, we investigated whether there was an excess or a deficit of heterozygotes.

#### Loss of genotype intensity

We had access to genotype intensity data for 243 RDCFIN bulls that had been genotyped with the Illumina BovineHD Genotyping BeadChip that included 725,293 autosomal SNPs [16]. We visually inspected the signal intensities around the QTL region using the *SVS8* program (Golden Helix) for genotyped bulls. Reduced intensities were observed between SNP BovineHD2300003056 (23:12,289,281, rs109948445) and SNP BOVINEHD2300003186 (23: 12,816,660, rs132859742). An animal was considered to carry the deletion if it met two conditions: the average signal intensity (‘Log_2_R ratio’) was less than 0, and 97 % of the SNPs were homozygous within the region defined by the signal intensities. Sires were categorized as carriers or noncarriers of the chromosomal deletion (CHR_DEL_), and the average intensity per SNP was calculated for both groups. An individual that carries CHR_DEL_ will be erroneously genotyped as homozygous for all loci within the deleted region because only one allele is present. Thus, a small aberration (3 %) was allowed, to account for possible genotyping errors.

### Search for recessive lethal haplotypes (absence of homozygotes) using 50 k genotype data

Absence of homozygotes for a common haplotype among live individuals is strong evidence for the presence of a recessive lethal allele [10]. Based on this, we searched for a recessive lethal allele on BTA23 segregating in Nordic Red dairy cattle. A total of 19,309 Nordic Red dairy cattle animals with 50 k genotypes, including 1119 SNPs on BTA23, were available. We discarded 136 SNPs that had a MAF less than 0.01 and 73 SNPs that had very strong deviations from HWP (*p* < 10^−5^). To avoid the influence of a deletion on phasing accuracy, we removed four SNPs that were located within the putative deleted region (between 12.2 and 12.9 Mb).

Next, we used Beagle 4.0 [31] to impute sporadic missing genotypes and to infer the haplotype phase. Finally, 901 SNPs remained on BTA23. To identify a haplotype that tagged the deleted region, we selected a 10-SNP region that spanned the putative deleted region (five SNPs upstream and five SNPs downstream) and covered a region between positions 11,818,761 and 13,122,318 bp. It was expected that one or more haplotypes in this window would be in tight linkage disequilibrium with the deleted region. If the deletion is recessive lethal, then the haplotype(s) that tag(s) it should only be present in the heterozygous condition; i.e. no live animal homozygous for the recessive lethal haplotype should be found.

## Results

### Genome scan for birth index

**Fig. 1:**
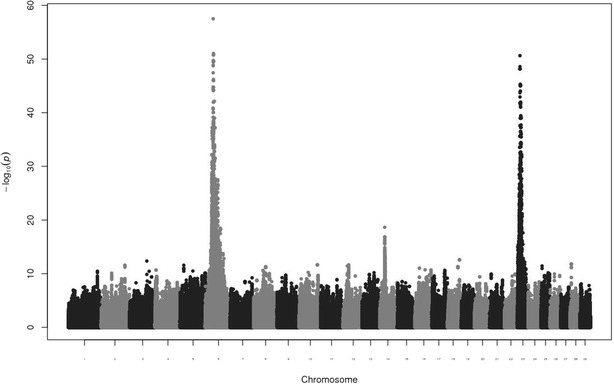
Manhattan plot for single-variant association analysis for birth index in Nordic Red dairy cattle. Association analyses were carried out using a sire model for whole-genome sequence variants

**Fig. 2:**
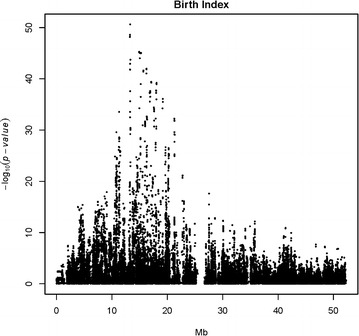
Association results for single-variant analysis, obtained using a sire model, for birth index on chromosome 23 in Nordic Red dairy cattle. The X-axis is chromosomal location in million base pairs and Y-axis is −log_10_(p)

Figure 1 presents the Manhattan plot for the QTL scan for BI based on WGS data in Nordic Red dairy cattle. Additional file 1: Table S1 provides the top associated genome-wide significant SNPs for each chromosome. Strong association signals for BI were observed on BTA6, 14 and 23. Previously, we reported a QTL for BI on BTA6 which increased calf size at birth and adult stature [32] and we attributed the increases in calving difficulties and stillbirth to the increased calf size at birth. In the present study, we focused on the QTL for BI that is located on BTA23. The most strongly associated SNP was located at 13,313,896 bp (rs722178836) and had an allele substitution effect of -0.72 additive genetic standard deviations and $- \log_{10} \left( {\text{p}} \right) = 50.63$ (Fig. 2).

### LMM analysis of the targeted region

**Fig. 3:**
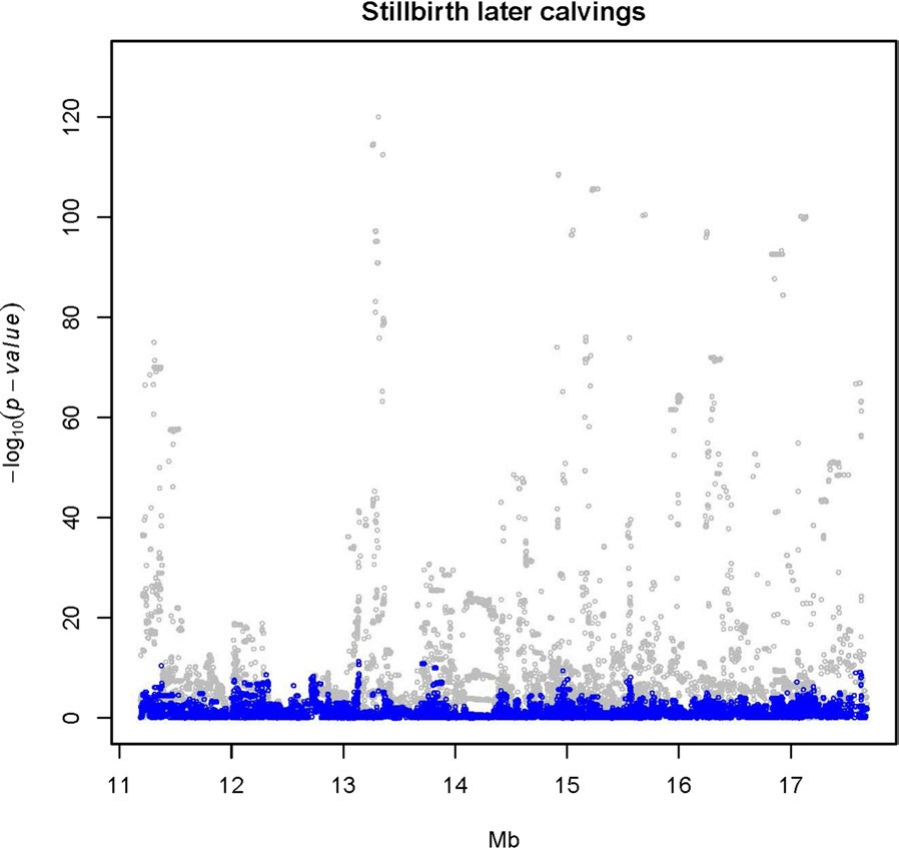
Single-variant association signal plots for calf survival at later calvings (SBL) (*light gray dots*) and single-variant analysis with the 10-SNP haplotype as a cofactor (*blue dots*). Haplotypes were constructed with the 10 top associated SNPs at the QTL peak and fitted as covariates in the model (*blue dots*)

**Fig. 4:**
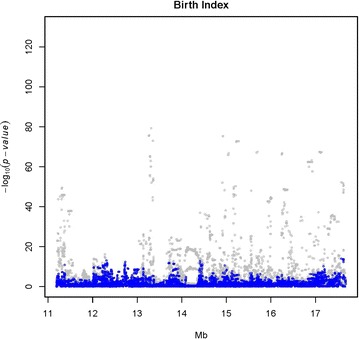
Single-variant association signal plots for birth index (BI) (*light gray dots*) and single-variant analysis with the 10-SNP haplotype as a cofactor (*blue dots*). Haplotypes were constructed with the 10 top associated SNPs at the QTL peak and fitted as covariates in the model (*blue dots*)

**Fig. 5:**
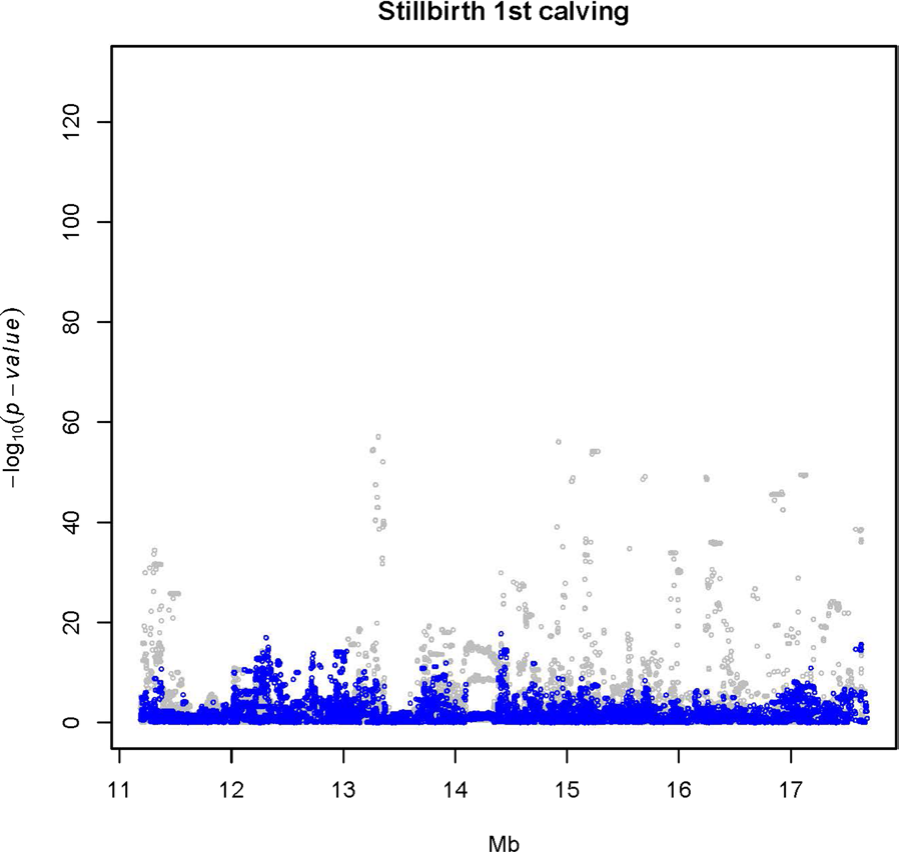
Single-variant association signal plots for calf survival at first calving (SBF) (*light gray dots*) and single-variant analysis with the 10-SNP haplotype as a cofactor (*blue dots*). Haplotypes were constructed with the 10 top associated SNPs at the QTL peak and fitted as covariates in the model (*blue dots*)

BI and three calving traits (SB, CE and CS) were analyzed for association with SNPs in the target region on BTA23 using a LMM. The SNPs that were most strongly associated with each trait are in Table 1. The strongest association signal was observed for SBL (light gray dots in Fig. 3), followed by BI (light gray dots in Fig. 4) and SBF (light gray dots in Fig. 5), with a peak at 13,313,896 bp (rs722178836). Association peaks for both CE and CS were positioned at 17.63 Mbp (see Additional file 2: Figures S1, S2). The $- \log_{10} \left( {\text{p}} \right)$ values were between 15.9 and 24.4, i.e. much smaller than those observed for SB and BI.

### Haplotype-based analysis of the targeted region

The strongest association signal among the analyzed traits was obtained for SBL (Table 1; Fig. 3). The 10 SNPs that were most strongly associated with SBL were used to construct a haplotype (Table 2). Diversity of these 10-SNP haplotypes was extremely low, with only five of the 1024 possible haplotypes being observed. Haplotypes and frequencies for the Nordic Red dairy cattle population were ATGTGAGTGA (0.9306), ATGTGAGTGC (0.0059), GCACAGCGAA (0.0019), GCACAGCGAC (0.0577) and GCACAGCGGA (0.0042).

When we analyzed haplotype as a random effect in the RHM, we observed that the haplotype GCACAGCGAC reduced the breeding values for SBL by 0.74 additive genetic standard deviations. The frequency of carriers of this haplotype (HAP_QTL_) was highest in RDCFIN (15.5 %), followed by RDCSWE (10.1 %) and RDCDNK (3.9 %). No homozygous individuals for this haplotype were observed. When this haplotype was included in the model as a cofactor, no additional significant associations were observed (blue dots in Figs. 3, 4, 5), suggesting that this haplotype could fully explain the QTL variance.

### Embryonic homozygosity for HAP_QTL_ is responsible for stillbirth

**Fig. 6:**
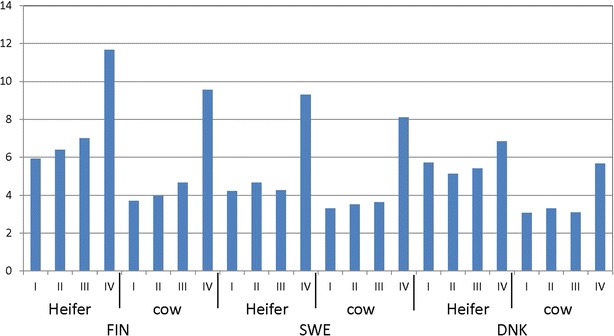
Stillbirth rate (%) by mating type according to the carrier status of the sire (S) and maternal grandsire (MGS) for the lethal haplotype (HAP_QTL_), based on the top 10 SNPs associated with stillbirth. *I* noncarrier S × MGS, *II* noncarrier S × carrier MGS, *III* carrier S × noncarrier MGS and *IV* carrier S × MGS matings, *FIN* Finland, *SWE* Sweden and *DNK* Denmark

We analyzed the stillbirth rate for the four mating types (I, II, III and IV) based on the carrier status of the sire and maternal grandsire. The stillbirth rate was approximately 6 % higher for matings in which both the sire and maternal grandsire carried the putative causative haplotype HAP_QTL_ compared to matings in which neither was a carrier (Fig. 6). We consistently observed higher calf mortality rates from carrier-sire by carrier-maternal grandsire matings across the three Nordic countries, in both the first and later calvings (Table 3).

### Presence of a large chromosomal deletion in the QTL region

#### Deviation from HWP

Significant deviations from HWP were observed for SNPs on the HD chip in the interval between 12.1 and 12.9 Mbp (see Additional file 3: Figure S3), all of which were due to an excess of homozygotes for the SNPs. This result provides a strong indication of a chromosomal deletion.

#### HD genotype intensity confirms the structural variation

**Fig. 7:**
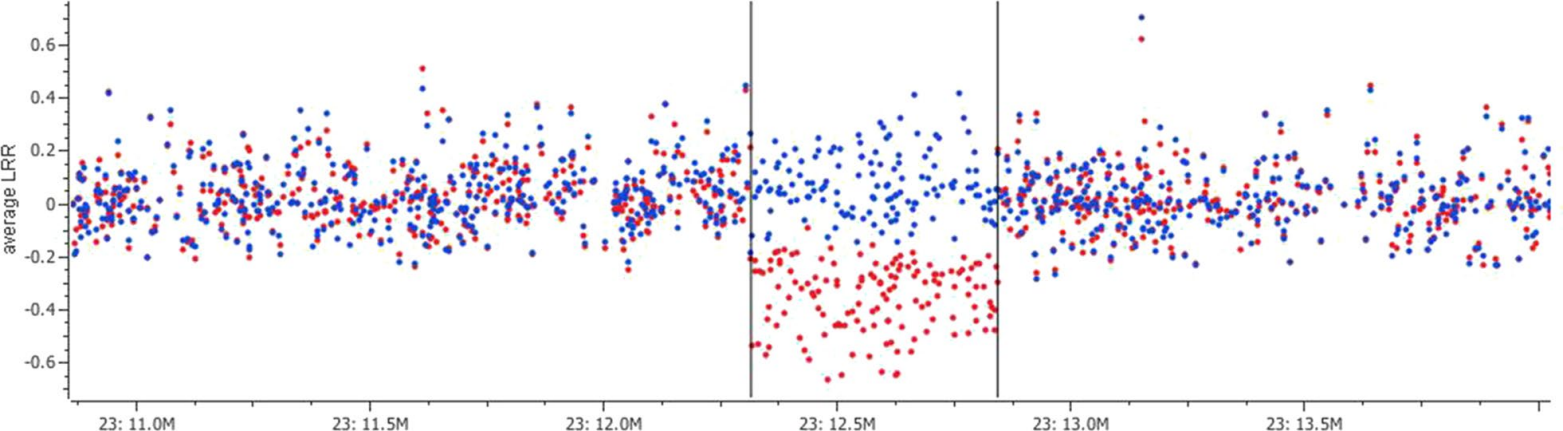
Average genotype signal intensity per SNP for carriers (*red*) and noncarriers (*blue*) of the chromosomal deletion (CHR_DEL_), plotted against their genomic positions. Carrier status of an individual was predicted based on loss of genotype intensity and homozygosity using SNPs from the Illumina BovineHD genotyping array

The plot of the average intensities from carriers and noncarriers of CHR_DEL_ (Fig. 7) showed a clear reduction in signal intensities for SNPs in the region between 12,289,281 and 12,816,660 bp, which confirmed the existence of a chromosomal deletion. Since the deleted region harbored several repetitive elements, it was difficult to identify the exact breaking points from the position of the split reads of the sequence data. Amplification across the deleted region was unsuccessful because unique primers for its extremities could not be designed. Sequence coverage increased in depth at the end region of the breaking point (see Additional file 4: Figure S4), which could be due to assembly problems or the presence of repetitive elements. These data, combined with the individual HD SNP intensity data and sequence alignments (results not shown), indicate that either the bovine genome assembly of this region is incorrect, or that the structural variation is more complex than just a simple deletion.

The deletion discovered here (CHR_DEL_) is not the only structural variation found in the region. Bickhart et al. [33] published a copy number variation (CNV) in Angus cattle that originated from Great Britain within the region affected by CHR_DEL_ (BTA23: 12,500,344–12,515,633 bp), and Hou et al. [34] found a CNV (BTA23: 12,938,090–13,081,504 bp) in Nordic Red dairy cattle near CHR_DEL_. Both CNV are gains of DNA sequence and are much smaller than the structural variation identified here.

**Fig. 8:**
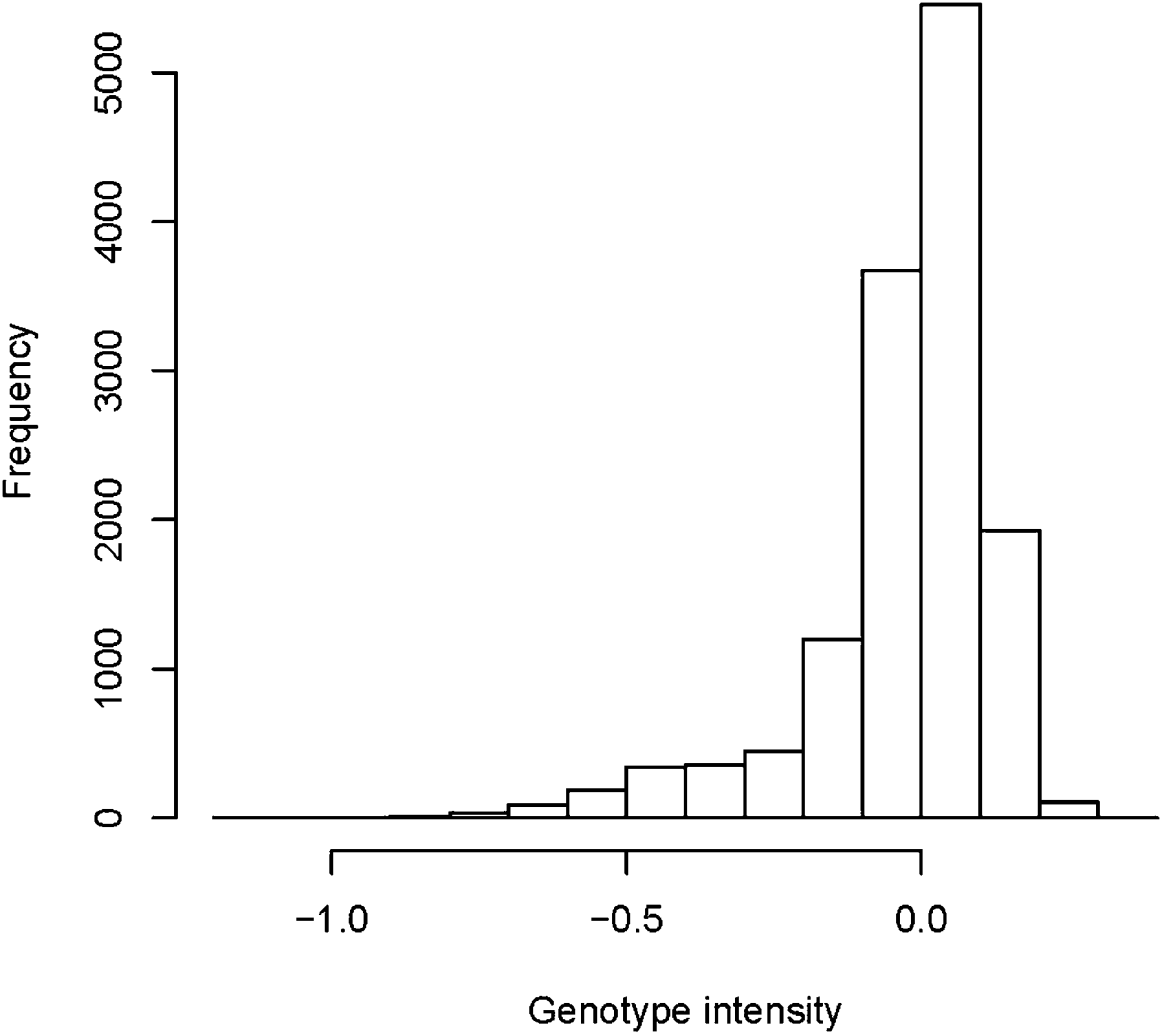
Density plot of genotype intensity for eight SNPs located within the chromosomal deletion in the Illumina BovineSNP50 BeadChip for an independent sample of Nordic Red dairy cattle

In addition, we checked the genotype intensity (log_2_R ratio) for eight SNPs on the 50 k chip located within the deleted region in a larger sample of 13,852 Nordic Red dairy cattle animals. The distribution of the intensities is in Fig. 8. Animals that were homozygous for all eight SNPs had low genotype intensities, which supports the existence of a chromosomal deletion. The length of this deletion was approximately 0.5 Mbp, as indicated by the HD intensity data and WGS depth.

## 50 k genotype data confirm the presence of a recessive lethal haplotype spanning the deleted region

We identified a haplotype (HAP_50k_) that spanned a region between 11,818,761 and 13,122,318 bp with a population frequency of 0.043, but we found no homozygous individuals for this haplotype. Under Hardy–Weinberg equilibrium, we would expect to have 36 homozygotes for this haplotype among the 19,309 50 k-genotyped animals. The probability of observing no homozygotes by chance is 2.31 × 10^−16^ (assuming a Poisson distribution), if this haplotype is not a recessive lethal variant. Absence of homozygotes for HAP_50k_ is strong evidence that Hap_50k_ either carries or tags a recessive lethal variant.

### Concordance of carriers of HAP_QTL_ and CHR_DEL_

#### WGS depth confirms the chromosomal deletion

We took advantage of the WGS information that was available for 83 Nordic Red dairy bulls. Six sequenced bulls carried the haplotype associated with stillbirth (HAP_QTL_). All six also showed an approximately halved sequencing depth between 12.29 and 12.84 Mbp (one example shown in Additional file 4: Figure S4), whereas such a reduction was not found for any of the noncarrier bulls. None of the 59 Holstein or Jersey bulls for which WGS data was analyzed, exhibited this reduction in sequencing depth for this region on BTA23.

#### CHR_DEL_ carriers inferred from HD genotype intensity

Among the 243 RDCFIN bulls with HD genotypes, 47 animals were declared carriers based on the criteria of an average genotype signal intensity less than 0 and homozygosity for more than 134 of the 139 loci within the putative deleted region between 12.2 and 12.8 Mb. Of these 47 carriers, 25 were homozygous for all 139 loci, 13 were homozygous for 138 loci, and nine were homozygous for 134–137 loci. We examined the concordance between carriers of CHR_DEL_ and carriers of the recessive lethal haplotype based on 50 k data (HAP_50k_). Among the 47 animals that carried the deletion, 42 carried the tagging haplotype. SNPs from the Illumina BovineSNP50 BeadChip located within the deleted region are in Additional file 5: Table S2.

## Discussion

A large-effect QTL for BI was detected on BTA23 in a genome-wide association study in Nordic Red dairy cattle. A detailed study of its component traits showed that it had a large effect on stillbirth. We constructed haplotypes from the 10 SNPs that were most strongly associated with stillbirth based on WGS data and identified animals that carried the haplotype that had a negative effect on stillbirth (HAP_QTL_). No homozygotes for HAP_QTL_ were observed. No other QTL effects were observed in the region when HAP_QTL_ was included in the model as a cofactor. Matings for which the sire and maternal grandsire carried HAP_QTL_ had significantly higher frequencies of stillbirth compared to matings for which the sire and/or maternal grandsire were a noncarrier.

We used the HD SNP genotypes to search for chromosomal deletions within the QTL region. Based on deviations from HWP and reduced genotype intensity, we observed a large chromosomal deletion (CHR_DEL_) and identified animals that carried this deletion. Using a large number of animals with 50 k genotypes, we confirmed the existence of a recessive lethal haplotype (HAP_50k_) that spanned the deleted region and identified carriers of HAP_50k_.

After studying the concordance between animals carrying HAP_QTL_, CHR_DEL_ and HAP_50k_, an approximately 500-kb deletion on BTA23 emerged as a strong candidate QTL for stillbirth. Two lines of evidence support this conclusion. First, among the 83 sequenced Nordic Red dairy cattle animals, the six bulls that carried HAP_QTL_ showed a window where read depth was reduced by 50 % compared to the rest of the genome. In contrast, no noncarrier bulls showed this reduction in sequencing depth. However, uncertainty remains on the exact position of the breakpoints of the deleted region due to the presence of other structural variants in this genomic region (see Additional file 4: Figure S4). Second, analysis of 50 k genotypes for a large number of individuals confirmed a recessive lethal haplotype (HAP_50k_) that spanned this deleted region.

Several observations support the conclusion that HAP_QTL_ is a recessive lethal haplotype that causes stillbirth. First, no homozygotes for HAP_QTL_ were observed. Second, the effect of HAP_QTL_ was observed in the case of carrier-sire and carrier-maternal grandsire matings for all three Nordic Red dairy cattle populations. There was a slight increase in stillbirths among type III matings in the Finnish population. This result may be due to a higher frequency of carriers of HAP_QTL_, such that the dam inherits the deletion from its dam (i.e. maternal granddam of the calf) even when the maternal grandsire is a noncarrier. Frequencies of carriers of HAP_QTL_ were much lower in the Swedish and Danish RDC populations. Consequently, no effect on fertility was observed in type III matings in these populations.

The increase in the rate of stillbirths among type IV matings was smaller than the 12.5 % expected for a fully penetrant recessive lethal allele. Because stillbirths are recorded by farmers, the difference may be due to some of the stillbirths not being reported to the central registration system. It is also possible that some deaths of calves occurred during late gestation and, thus, were not recorded by farmers as stillbirths. The stillbirth is recorded as a binary trait but we analyzed it by assuming a normal distribution to test the effect of mating type on calf survival at birth. A logistic regression model might have been a better choice for the analysis of this phenotype.

### Genes located within the deleted region

**Fig. 9:**
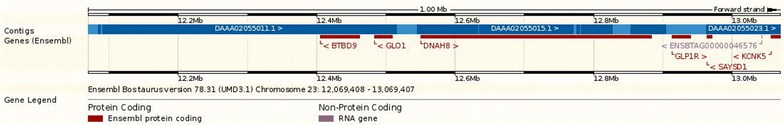
Genes located within the targeted region based on the Ensembl *Bos taurus* version 78 (UMD3.1, http://www.ensembl.org/)

Three genes are located within the deleted region: (1) *BTB (POZ) domain containing 9* (*BTBD9*, 12,405,340–12,462,065 bp), (2) *glyoxalase I* (*GLO1*, 12,483,468–12,509,232 bp) and (3) *dynein, axonemal, heavy chain 8* (*DNAH8*, 12,550,367–12,883,939 bp) (Fig. 9). Which, if any, of these genes causes stillbirth if deleted is not obvious from their known gene functions. Phenotypes of *BTBD9* knockout mice suggest that BTBD9 is involved in synaptic plasticity, learning, memory and protein alterations associated with vesicle recycling and endocytosis [35]. When glyoxalase I activity decreased in situ through aging and oxidative stress, levels of glycation and tissue damage increased. These effects may be associated with a risk of developing vascular complications due to diabetes and uremia [36]. Overexpression of glyoxalase 1 in bone marrow cells reversed defective neovascularization in streptozotocin-induced diabetic mice [37]. Diseases associated with DNAH8 include myosin storage myopathy and reduced body myopathy (http://www.genecards.org/cgi-bin/carddisp.pl?gene=DNAH8, accessed 23 December 2014). The most well-known disease associated with dynein malfunction in humans is polycystic kidney disease (PCD; also known as Kartagener syndrome), which is characterized by malfunctioning of the cilia and sperm, as well as respiratory problems. Around half of the PCD patients also have *situs inversus*. Extreme defects in laterality may be diagnosed as heterotaxy with asplenia or polysplenia [38].

The Arachnomelia mutation described by Buitkamp et al. [39], located near the QTL peak, was not the cause of the stillbirth cases described here. Arachnomelia syndrome in Simmental cattle is caused by a 2-bp deletion at the *MOCS1* locus. This deletion was not observed among the sequenced RDC bulls. Haplotypes constructed around the *MOCS1* locus (HAP_MOCS1_) did not show any association with stillbirth in Nordic Red dairy cattle (see Additional file 6: Figure S5).

### No other indexes in the Nordic breeding evaluation showed significant QTL near the deleted region

We carried out association studies for the other indexes included in the breeding goal for the Nordic Red dairy cattle breed (separate studies, details not presented here). The QTL for stillbirth on BTA23 was genome-wide-significant only for body conformation index, with a peak at 12,153,108 bp (rs110295087, $- \log_{10} \left( {\text{p}} \right) = 13.92$). The highest peak for body conformation on BTA23 was at 40,658,187 bp (rs133817421, $- \log_{10} \left( {\text{p}} \right) = 17.98$). No other indexes measured in Nordic Red dairy cattle showed genome-wide-significant QTL in this region. Thus, we found no evidence that this QTL for stillbirth exhibits pleiotropic effects on other traits in the breeding goal.

Kadri et al. [16] reported very high frequencies of carriers (13 to 32 %) of a large deletion on BTA12 that has a recessive lethal effect and segregates in three Nordic Red dairy cattle populations. They suggested that these high frequencies were due to the large positive effects on milk production traits. In contrast, low to moderate frequencies of carriers of HAP_QTL_ indicate that this haplotype has not been actively selected by hitchhiking such as the deletion on BTA12 in the Nordic Red cattle.

We used two imputation software packages, Impute2 and Beagle to impute 50 k genotypes to HD and thereafter to whole-genome sequence variants, respectively. These two imputations were performed in different projects. The performances of both software packages are very similar [40] and therefore, we did not expect to find any influence of using two software packages on our results. It is theoretically expected that a method that combines linkage information and LD at the population level is more efficient for genotype imputation and haplotype phasing in cattle for which good pedigree records are available; however, several studies have not confirmed this hypothesis (e.g. Ma et al. [41]).

We are surprised that in spite of its moderate frequency in some Nordic Red dairy cattle populations, this deletion was undetected, to date. This may be due to the lack of any obvious physical deformities of the dead calves. Examination of a calf that is homozygous for the deleted region may help to identify the gene for which loss of function constitutes the proximate cause of stillbirth. However, our efforts to obtain carcasses of dead calves from carrier–carrier matings have so far been unsuccessful.

## Conclusions

In a whole-genome association scan for Nordic Red dairy cattle, we detected a QTL on BTA23 that affects BI. We dissected this QTL by analyzing the component traits of BI, observed that it had a very large effect on stillbirth and fine-mapped the QTL. We present evidence that the underlying causative variant acts as a recessive lethal variant. Based on analyses of WGS data of carrier and noncarrier individuals for HAP_QTL_, an approximately 0.5-Mbp deletion/structural variation emerged as a strong candidate responsible for stillbirth.

## Availability of supporting data

The sequence data of the animals are publicly available in the Sequence read Archive of NCBI (http://www.ncbi.nlm.nih.gov/sra) under accession numbers SRX527690-SRX527732 and are part of the 1000 bull genomes project (http://www.1000bullgenomes.com).

## Additional files

10.1186/s12711-016-0215-z Top associated SNPs on bovine autosomes, if genome-wide significant in the genome scan for birth index in Nordic red cattle. The most significantly associated SNPs with birth index on each chromosome from the genome are listed.
10.1186/s12711-016-0215-z Linear mixed model analysis for calf size at first (A) and later calvings (B) for the targeted QTL region on bovine chromosome 23. Association results for calf size for the targeted region. **Figure S2.** Linear mixed model analysis for calving ease at first (A) and later calvings (B) for the targeted QTL region on bovine chromosome 23. Association results for calf size for the targeted region.
10.1186/s12711-016-0215-z Test for Hardy-Weinberg proportions for the SNPs within the targeted region on bovine chromosome 23. Red circle indicates an excess of homozygotes and green circle indicates a deficit of homozygotes.
10.1186/s12711-016-0215-z Sequence coverage from a non-carrier and a carrier bull of HAP_QTL_. The red rectangle shows the end region of the breaking point. The coverage peaks located in the end region could be due to assembly problems or to repetitive elements.
10.1186/s12711-016-0215-z SNPs included in a custom-made low-density SNP chip to identify carriers for the 500 kb deletion. SNPs located within the putative chromosomal deletion on bovine chromosome 23 were included in a custom-made low-density SNP chip to identify carriers based on genotype intensity from SNP array binding.
10.1186/s12711-016-0215-z Plots of association signals for single marker analysis (gray), single marker analysis with the 8-SNP haplotype (located within the *MOCS1* gene) as cofactor (blue) for calf survival (latter calvings). The position of the *MOCS1* gene is marked by a red rectangle. The haplotype could not explain the QTL variance for the targeted region.

## References

[1] Agerholm JS, Bendixen C, Andersen O, Arnbjerg J (2001). Complex vertebral malformation in holstein calves. J Vet Diagn Invest.

[2] Agerholm JS, McEvoy F, Arnbjerg J (2006). Brachyspina syndrome in a Holstein calf. J Vet Diagn Invest.

[3] el-Hamidi M, Leipold HW, Vestweber JG, Saperstein G (1989). Spinal muscular atrophy in Brown Swiss calves. Zentralbl Veterinarmed A.

[4] Shanks RD, Robinson JL (1989). Embryonic mortality attributed to inherited deficiency of uridine monophosphate synthase. J Dairy Sci.

[5] Shuster DE, Kehrli ME, Ackermann MR, Gilbert RO (1992). Identification and prevalence of a genetic defect that causes leukocyte adhesion deficiency in Holstein cattle. Proc Natl Acad Sci USA.

[6] Leipold HW, Huston K, Barton EP, Holman JR, Troyer DL (1990). Rectovaginal constriction in Jersey cattle: genetics and breed dynamics. J Dairy Sci.

[7] Leipold HW, Blaugh B, Huston K, Edgerly CG, Hibbs CM (1973). Weaver syndrome in Brown Swiss cattle: clinical signs & pathology. Vet Med Small Anim Clinic.

[8] Lamb RC, Arave CW, Shupe JL (1976). Inheritance of limber legs in Jersey cattle. J Hered.

[9] Drögemuller C, Tetens J, Sigurdsson S, Gentile A, Testoni S, Lindblad-Toh K (2010). Identification of the bovine Arachnomelia mutation by massively parallel sequencing implicates sulfite oxidase (SUOX) in bone development. PLoS Genet.

[10] VanRaden PM, Olson KM, Null DJ, Hutchison JL (2011). Harmful recessive effects on fertility detected by absence of homozygous haplotypes. J Dairy Sci.

[11] Fritz S, Capitan A, Djari A, Rodriguez SC, Barbat A, Baur A (2013). Detection of haplotypes associated with prenatal death in dairy cattle and identification of deleterious mutations in *GART*, *SHBG* and *SLC37A2*. PLoS One.

[12] Sahana G, Nielsen US, Aamand GP, Lund MS, Guldbrandtsen B (2013). Novel harmful recessive haplotypes identified for fertility traits in Nordic Holstein cattle. PLoS One.

[13] Pausch H, Schwarzenbacher H, Burgstaller J, Flisikowski K, Wurmser C, Jansen S (2015). Homozygous haplotype deficiency reveals deleterious mutations compromising reproductive and rearing success in cattle. BMC Genom.

[14] Daetwyler HD, Capitan A, Pausch H, Stothard P, van Binsbergen R, Brondum RF (2014). Whole-genome sequencing of 234 bulls facilitates mapping of monogenic and complex traits in cattle. Nat Genet.

[15] Schulman NF, Sahana G, Iso-Touru T, McKay SD, Schnabel RD, Lund MS (2011). Mapping of fertility traits in Finnish Ayrshire by genome-wide association analysis. Anim Genet.

[16] Kadri NK, Sahana G, Charlier C, Iso-Touru T, Guldbrandtsen B, Karim L (2014). A 660-Kb deletion with antagonistic effects on fertility and milk production segregates at high frequency in Nordic Red cattle: additional evidence for the common occurrence of balancing selection in livestock. PLoS Genet.

[17] Sieber M, Freeman AE, Kelley DH (1989). Effects of body measurements and weight on calf size and calving difficulty of Holsteins. J Dairy Sci.

[18] Garrick DJ, Taylor JF, Fernando RL (2009). Deregressing estimated breeding values and weighting information for genomic regression analyses. Genet Sel Evol.

[19] Boelling D, Sander Nielsen U, Pösö J, Erikson JÅ, Aamand GP (2007). Genetic evaluation of calving traits in Denmark, Finland, and Sweden. Interbull Bull.

[20] Zimin AV, Delcher AL, Florea L, Kelley DR, Schatz MC, Puiu D (2009). A whole-genome assembly of the domestic cow, *Bos taurus*. Genome Biol.

[21] Howie BN, Donnelly P, Marchini J (2009). A flexible and accurate genotype imputation method for the next generation of genome-wide association studies. PLoS Genet.

[22] Browning BL, Browning SR (2009). A unified approach to genotype imputation and haplotype-phase inference for large data sets of trios and unrelated individuals. Am J Hum Genet.

[23] Brøndum RF, Guldbrandtsen B, Sahana G, Lund MS, Su G (2014). Strategies for imputation to whole genome sequence using a single or multi-breed reference population in cattle. BMC Genomics.

[24] Höglund JK, Sahana G, Brondum RF, Guldbrandtsen B, Buitenhuis B, Lund MS (2014). Fine mapping QTL for female fertility on BTA04 and BTA13 in dairy cattle using HD SNP and sequence data. BMC Genomics.

[25] Sahana G, Guldbrandtsen B, Thomsen B, Holm LE, Panitz F, Brøndum RF (2014). Genome-wide association study using high-density single nucleotide polymorphism arrays and whole-genome sequences for clinical mastitis traits in dairy cattle. J Dairy Sci.

[26] Madsen P, Jensen J, Labouriau R, Christensen OF, Sahana G. DMU—A package for analyzing multivariate mixed models in quantitative genetics and genomics. In Proceedings of the: 10th world congress of genetics applied to livestock production: 17–22 August 2014; Vancouver. 2014. https://asas.org/docs/default-source/wcgalp-posters/699_paper_9580_manuscript_758_0.pdf?sfvrsn=2.

[27] Yu J, Pressoir G, Briggs WH, Vroh Bi I, Yamasaki M, Doebley JF (2006). A unified mixed-model method for association mapping that accounts for multiple levels of relatedness. Nat Genet.

[28] Boleckova J, Christensen OF, Sorensen P, Sahana G (2012). Strategies for haplotype-based association mapping in a complex pedigreed population. Czech J Anim Sci.

[29] Kadri NK, Sahana G, Guldbrandtsen B, Lund MS, Druet T (2014). Efficiency of haplotype-based methods to fine-map QTLs and embryonic lethal variants affecting fertility: illustration with a deletion segregating in Nordic Red cattle. Livest Sci.

[30] Charlier C, Agerholm JS, Coppieters W, Karlskov-Mortensen P, Li W, de Jong G (2012). A deletion in the bovine *FANCI* gene compromises fertility by causing fetal death and brachyspina. PLoS One.

[31] Browning SR, Browning BL (2007). Rapid and accurate haplotype phasing and missing-data inference for whole-genome association studies by use of localized haplotype clustering. Am J Hum Genet.

[32] Sahana G, Höglund JK, Guldbrandtsen B, Lund MS (2015). Loci associated with adult stature also affect calf birth survival in cattle. BMC Genet.

[33] Bickhart DM, Hou Y, Schroeder SG, Alkan C, Cardone MF, Matukumalli LK (2012). Copy number variation of individual cattle genomes using next-generation sequencing. Genome Res.

[34] Hou YL, Liu GE, Bickhart DM, Cardone MF, Wang K, Kim ES (2011). Genomic characteristics of cattle copy number variations. BMC Genomics.

[35] DeAndrade MP, Zhang L, Doroodchi A, Yokoi F, Cheetham CC, Chen HX (2012). Enhanced hippocampal long-term potentiation and fear memory in Btbd9 mutant mice. PLoS One.

[36] Thornalley PJ (2003). Glyoxalase I—structure, function and a critical role in the enzymatic defence against glycation. Biochem Soc Trans.

[37] Vulesevic B, McNeill B, Geoffrion M, Kuraitis D, McBane JE, Lochhead M (2014). Glyoxalase-1 overexpression in bone marrow cells reverses defective neovascularization in STZ-induced diabetic mice. Cardiovasc Res.

[38] Burton EC, Olson M, Rooper L (2014). Defects in laterality with emphasis on heterotaxy syndromes with asplenia and polysplenia: an autopsy case series at a single institution. Pediatr Dev Pathol.

[39] Buitkamp J, Semmer J, Götz KU (2011). Arachnomelia syndrome in Simmental cattle is caused by a homozygous 2-bp deletion in the *molybdenum cofactor synthesis step 1* gene (*MOCS1*). BMC Genet.

[40] Marchini J, Howie B (2010). Genotype imputation for genome-wide association studies. Nat Rev Genet.

[41] Ma P, Brøndum RF, Zhang Q, Lund MS, Su G (2013). Comparison of different methods for imputing genome-wide marker genotypes in Swedish and Finnish Red Cattle. J Dairy Sci.

