# Supplementary material for: A 0.5-Mbp deletion on bovine chromosome 23 is a strong candidate for stillbirth in Nordic Red cattle
Source: Genet Sel Evol. 2016 Apr 18;48:35. doi: 10.1186/s12711-016-0215-z (PMC4835938; doi:10.1186/s12711-016-0215-z)
Supplement: Supplementary file 1 — 10.1186/s12711-016-0215-z Top associated SNPs on bovine autosomes, if genome-wide significant in the genome scan for birth index in Nordic red cattle. The most significantly associated SNPs with birth index on each chromosome from the genome are listed. [file 12711_2016_215_MOESM1_ESM.docx]

**Table S1.** The top SNP from each chromosome, if genome-wide significant from genome scan of birth index in Nordic red cattle.

| SNP (Chromosome: position in bp) | Minor allele frequency | Allele substitution effect | Standard error | -log_10_(p) |
| --- | --- | --- | --- | --- |
| Chr1:139973867 | 0.014 | -6.53 | 0.98 | 10.45 |
| Chr2:113978228 | 0.337 | -1.65 | 0.24 | 11.55 |
| Chr3:82529160 | 0.238 | 1.92 | 0.26 | 12.36 |
| Chr4:5249468 | 0.339 | 1.52 | 0.23 | 10.67 |
| Chr5:18105417 | 0.043 | 4.03 | 0.57 | 11.58 |
| Chr6:38127504 | 0.312 | -3.77 | 0.23 | 57.50 |
| Chr7:105694348 | 0.016 | -5.57 | 0.90 | 9.24 |
| Chr8:58450067 | 0.023 | -5.77 | 0.83 | 11.30 |
| Chr9:41272526 | 0.036 | -3.76 | 0.59 | 9.69 |
| Chr10:87993868 | 0.032 | -4.89 | 0.69 | 11.66 |
| Chr11:77663635 | 0.352 | 1.39 | 0.23 | 8.88 |
| Chr12:26810334 | 0.452 | 1.56 | 0.22 | 11.65 |
| Chr13:57530105 | 0.108 | -2.37 | 0.36 | 10.19 |
| Chr14:24656389 | 0.195 | 2.48 | 0.27 | 18.64 |
| Chr15:23622699 | 0.494 | -1.36 | 0.23 | 8.52 |
| Chr16:21450014 | 0.059 | -3.46 | 0.51 | 11.02 |
| Chr17:61944623 | 0.065 | -3.12 | 0.47 | 10.63 |
| Chr18:57517094 | 0.035 | 4.68 | 0.64 | 12.60 |
| Chr20:24585455 | 0.153 | -2.22 | 0.35 | 9.44 |
| Chr21:7884339 | 0.044 | -4.17 | 0.65 | 9.91 |
| Chr22:50867420 | 0.251 | -1.71 | 0.27 | 9.94 |
| Chr23:13313896 | 0.059 | -7.17 | 0.47 | 50.63 |
| Chr24:53893876 | 0.131 | -1.98 | 0.33 | 8.48 |
| Chr25:4567770 | 0.056 | -3.40 | 0.49 | 11.44 |
| Chr26:21108033 | 0.167 | -1.97 | 0.30 | 9.95 |
| Chr28:5299056 | 0.221 | -1.93 | 0.27 | 11.83 |
