# Supplementary material for: A 0.5-Mbp deletion on bovine chromosome 23 is a strong candidate for stillbirth in Nordic Red cattle
Source: Genet Sel Evol. 2016 Apr 18;48:35. doi: 10.1186/s12711-016-0215-z (PMC4835938; doi:10.1186/s12711-016-0215-z)
Supplement: Supplementary file 2 — 10.1186/s12711-016-0215-z Linear mixed model analysis for calf size at first (A) and later calvings (B) for the targeted QTL region on bovine chromosome 23. Association results for calf size for the targeted region. Figure S2. Linear mixed model analysis for calving ease at first (A) and later calvings (B) for the targeted QTL region on bovine chromosome 23. Association results for calf size for the targeted region. [file 12711_2016_215_MOESM2_ESM.docx]

**Figure S1.** Linear mixed model analysis for calf size at first (A) and later calvings (B) for the targeted QTL region on chromosome 23.

**A**

 **Supplementary**

**B**

**Figure S2.** Linear mixed model analysis for calving ease at first (A) and later calvings (B) for the targeted QTL region on chromosome 23

**A**

**B**
