# Supplementary material for: A 0.5-Mbp deletion on bovine chromosome 23 is a strong candidate for stillbirth in Nordic Red cattle
Source: Genet Sel Evol. 2016 Apr 18;48:35. doi: 10.1186/s12711-016-0215-z (PMC4835938; doi:10.1186/s12711-016-0215-z)

**Figure S3.** Test for hardy-Weinberg proportions for the SNPs within the targeted region on chromosome 23; Red: excess of homozygotes, green: deficiency of homozygotes.


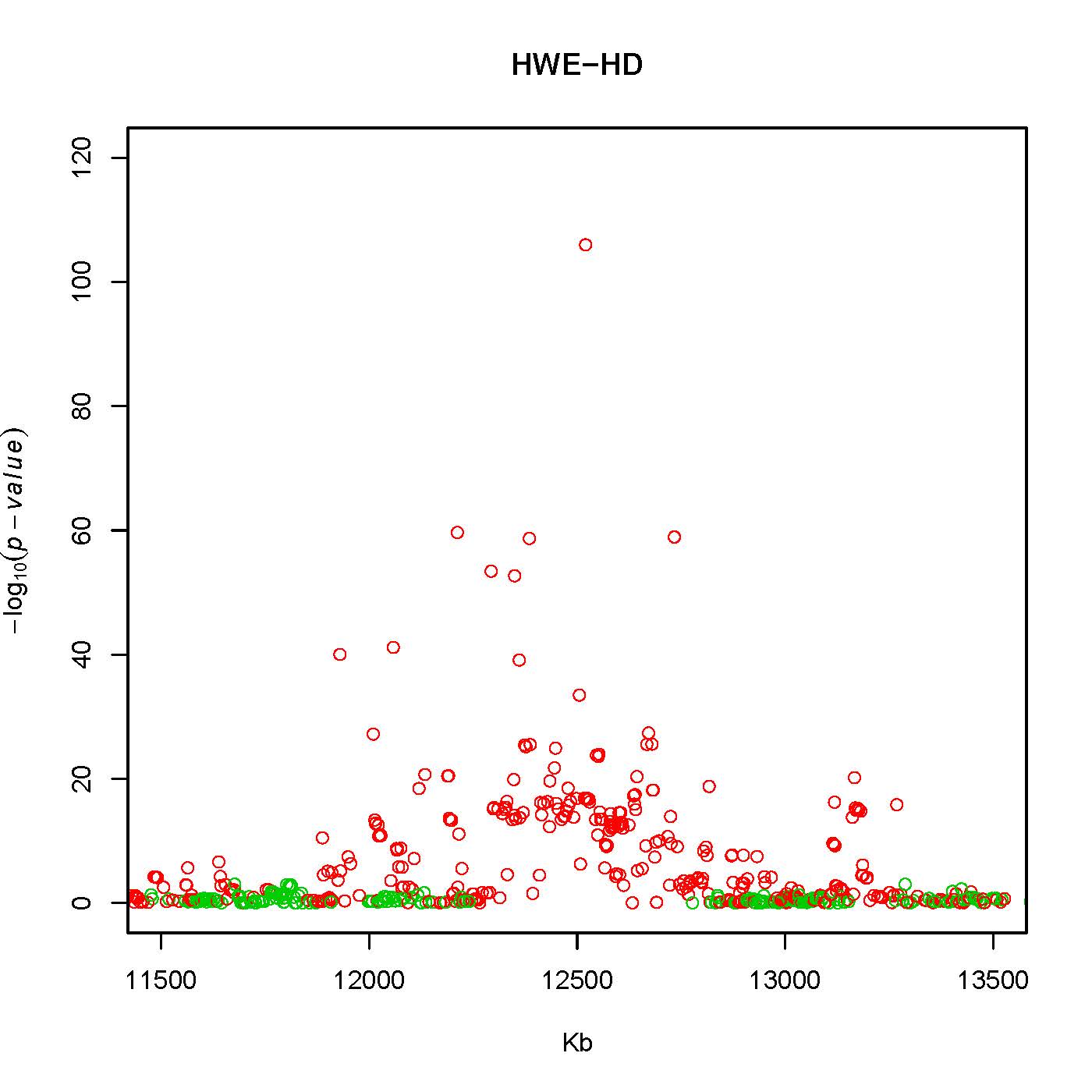

Supplement: Supplementary file 3 — 10.1186/s12711-016-0215-z Test for Hardy-Weinberg proportions for the SNPs within the targeted region on bovine chromosome 23. Red circle indicates an excess of homozygotes and green circle indicates a deficit of homozygotes. [file 12711_2016_215_MOESM3_ESM.docx]
