# Supplementary material for: A 0.5-Mbp deletion on bovine chromosome 23 is a strong candidate for stillbirth in Nordic Red cattle
Source: Genet Sel Evol. 2016 Apr 18;48:35. doi: 10.1186/s12711-016-0215-z (PMC4835938; doi:10.1186/s12711-016-0215-z)

**Figure S4.** Sequence coverage from a non-carrier and a carrier bull of HAP_QTL._ Red rectangle shows the end region of the breaking point.


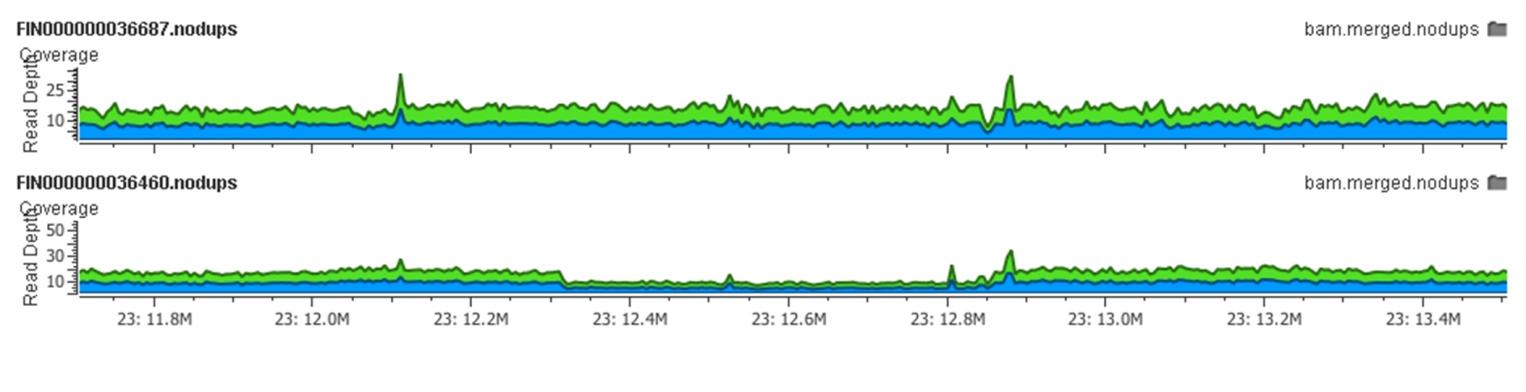

Supplement: Supplementary file 4 — 10.1186/s12711-016-0215-z Sequence coverage from a non-carrier and a carrier bull of HAPQTL. The red rectangle shows the end region of the breaking point. The coverage peaks located in the end region could be due to assembly problems or to repetitive elements. [file 12711_2016_215_MOESM4_ESM.docx]
