# Supplementary material for: A 0.5-Mbp deletion on bovine chromosome 23 is a strong candidate for stillbirth in Nordic Red cattle
Source: Genet Sel Evol. 2016 Apr 18;48:35. doi: 10.1186/s12711-016-0215-z (PMC4835938; doi:10.1186/s12711-016-0215-z)
Supplement: Supplementary file 5 — 10.1186/s12711-016-0215-z SNPs included in a custom-made low-density SNP chip to identify carriers for the 500 kb deletion. SNPs located within the putative chromosomal deletion on bovine chromosome 23 were included in a custom-made low-density SNP chip to identify carriers based on genotype intensity from SNP array binding. [file 12711_2016_215_MOESM5_ESM.docx]

**Table S2.** SNPs included in a custom-made low-density SNP chip to identify carriers for the 500 kb deletion

| Reference-ID | Position (BP) | SNP_name |
| --- | --- | --- |
| rs29027218 | 12343498 | Hapmap54829-rs29027218 |
| rs41586721 | 12579560 | BTA-55455-no-rs |
| rs41586723 | 12447484 | Hapmap44604-BTA-55457 |
| rs41619834 | 12384489 | Hapmap39973-BTA-119855 |
| rs41631512 | 12519690 | Hapmap49110-BTA-29232 |
| rs108943824 | 12753112 | ARS-BFGL-NGS-22142 |
| rs109103219 | 12548841 | ARS-BFGL-BAC-30064 |
| rs109863788 | 12663012 | Hapmap30236-BTA-137040 |
| rs109939328 | 12472891 | ARS-BFGL-NGS-102552 |
