# Supplementary material for: A 0.5-Mbp deletion on bovine chromosome 23 is a strong candidate for stillbirth in Nordic Red cattle
Source: Genet Sel Evol. 2016 Apr 18;48:35. doi: 10.1186/s12711-016-0215-z (PMC4835938; doi:10.1186/s12711-016-0215-z)
Supplement: Supplementary file 6 — 10.1186/s12711-016-0215-z Plots of association signals for single marker analysis (gray), single marker analysis with the 8-SNP haplotype (located within the MOCS1 gene) as cofactor (blue) for calf survival (latter calvings). The position of the MOCS1 gene is marked by a red rectangle. The haplotype could not explain the QTL variance for the targeted region. [file 12711_2016_215_MOESM6_ESM.docx]

**Figure S5.** Plots of association signals for single marker analysis (gray), single marker analysis with the 8-SNP haplotype (located within the *MOCS1* gene) as cofactor (blue) for calf survival (later calvings)

 
